# The role of correlated factors based on Pender health promotion model in brushing behavior in the 13–16 years old students of Guilan, Iran

**DOI:** 10.1186/s13052-021-01063-y

**Published:** 2021-05-12

**Authors:** Robabeh Haghi, Asieh Ashouri, Mahmood Karimy, Nooshin Rouhani-Tonekaboni, Parisa Kasmaei, Farahnaz Pakdaman, Iraj Zareban

**Affiliations:** 1 Department of Health Education and Promotion, School of Health, Guilan University of Medical Sciences, Rasht, Iran; 2 Department of Cardiology, Cardiovascular Diseases Research CenterHeshmat Hospital, School of Medicine, Guilan University of Medical Sciences, Rasht, Iran; 3 Social Determinants of Health Research Center, Saveh University of Medical Sciences, Saveh, Iran; 4 Department of Health Education and Promotion, Research Center of Health and Environment, School of Health, Guilan University of Medical Sciences, 41857-33411, Realat St., Rasht, Iran; 5 Department of Elderly and Middle-aged Health, Vice-chancellor of Health, Guilan University of Medical Sciences, Rasht, Iran; 6 Health Promotion Research Center, Zahedan University of Medical Science, Zahedan, Iran

**Keywords:** Brushing behavior, Students, Health promotion Model (HPM)

## Abstract

**Background:**

Tooth decay is one of the diseases that is closely related to people’s behaviors and it can have adverse effects on their performance and their success in the future. Brushing twice a day is the simplest and most effective way to reduce tooth decay. The study aim was to determining the roles of correlational factors based on the Pender’s health promotion model in brushing behavior of ninth grade students at urban public schools of Guilan province during the academic year 2019.

**Methods:**

The present study was cross-sectional and had a descriptive-analytical type. We performed the multi-stage random sampling on 761 ninth-grade students (374 girls and 387 boys) at urban public schools of six counties (ten cities) of Guilan province in 2019. The primary tool was a questionnaire on oral health behaviors focusing on brushing behavior. In the present study, which was conducted only on brushing behavior, we revised and changed the initial questionnaire during the sessions of the research team, and then confirmed its validity and reliability. The questionnaire consisted of three parts, demographic characteristics, constructs of health promotion model, and brushing behavior. We analyzed data in SPSS 21 using regression models.

**Results:**

20.1% of students brushed their teeth at least twice a day. Optimal behavior had a statistically significant relationship with parents’ education level, and gender. Based on the regression model, the health promotion model constructs described 58% of the variance of the commitment to plan of action. Perceived self-efficacy, situational influences, and perceived barriers of action had significantly stronger relationships with commitment to plan of action respectively. Multiple logistic regression analysis indicated that one-unit increase in scores of commitment to plan of action and self-efficacy increased the chance of desired behavior by 12 and 14% respectively.

**Conclusion:**

Due to the low rate of brushing behavior in the students and the predictive power of the health promotion model in brushing behavior, we suggest planning and implementation of educational interventions for this group with an emphasis on influencing the commitment to plan of action, self-efficacy, and also the level of knowledge.

**Supplementary Information:**

The online version contains supplementary material available at 10.1186/s13052-021-01063-y.

## Background

The World Health Organization (WHO) considers oral health as a necessity and part of public health in life and indicates that low oral health can have a profound effect on quality of life and affects the overall health of humans [1]. Oral and dental diseases, especially dental caries and periodontal diseases, are more common in the world [2]. When tooth decay goes untreated, it can lead to pain. In deep carious lesions, the risk of pulp involvement and abscesses increases, which usually causes swelling and discomfort when chewing. Candida, osteomyelitis, or sepsis due to dental infection leading to hospitalization have been reported in countries with poor access to dental services [1, 3].

Using a toothbrush is the first and most practical and common way to clean teeth [4]. Self-care recommendations emphasize brushing twice a day (optimal behavior) [5]. Based on the (Childhood and Adolescence Surveillance and Prevention of Adult Non-communicable disease study) Caspian study in Iran, 23.1% of students in Iran brush their teeth more than once a day, and the lowest and highest rates were in Kerman (16.66%) and Kermanshah (33.75%) respectively. The rate was 23.5% in Guilan students [6].

Adolescence is a difficult and challenging transition from childhood dependence to the independence and responsibility of youth and adulthood [7, 8]. Emphasizing adolescents’ responsibilities without any coercion can help them accept their new role [8]. Identifying the effective factors on oral health behaviors in students and as a result of this, designing appropriate educational interventions, is necessary to improve these behaviors [3].Previous studies found that pay attention to prevention and oral health in students is a key strategy to promote oral health in society, and the first step in prevention and oral health of students is to identify the determinants of their behavior [8, 9].

**Fig. 1:**
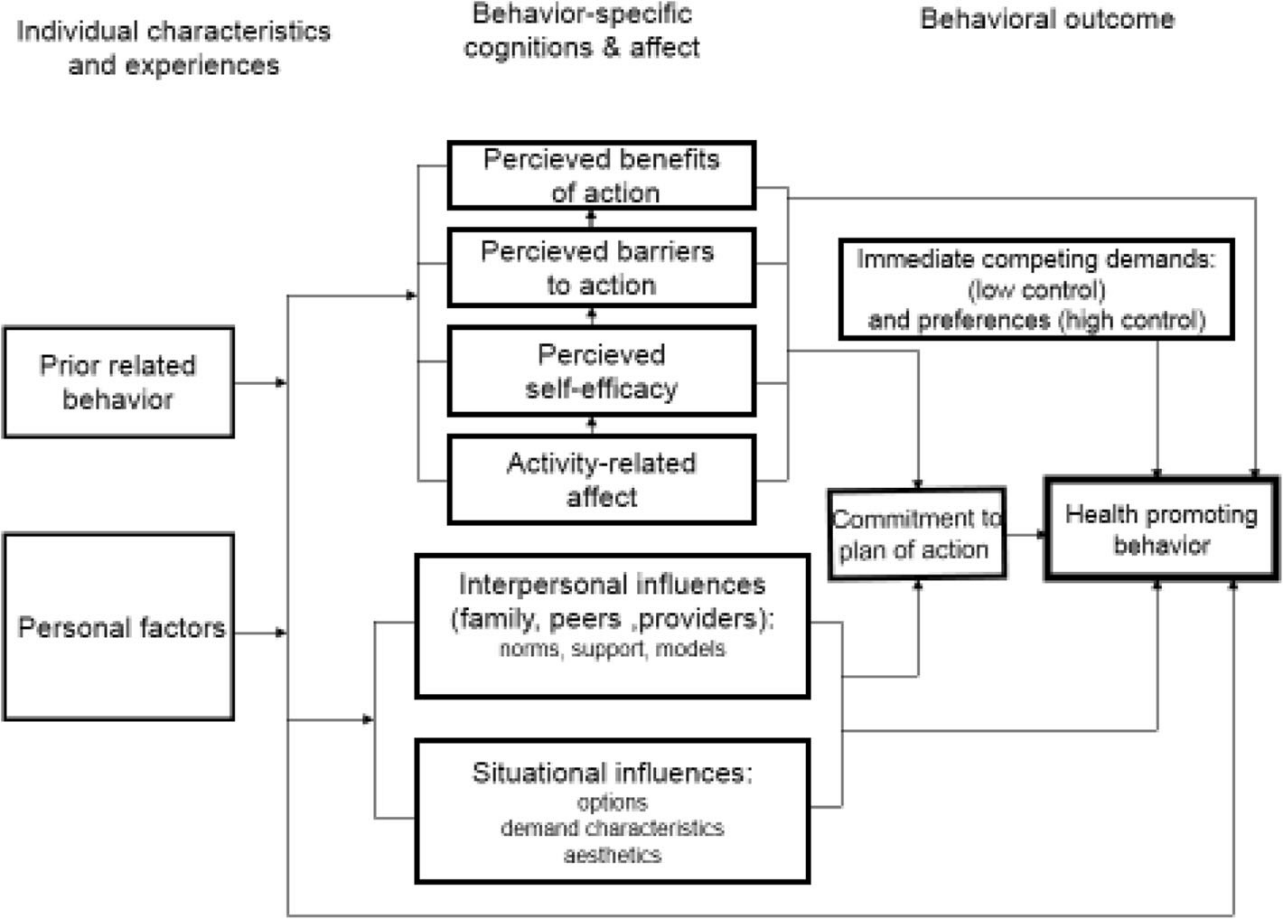
Pender’s health promotion model

Behavioral and health experts use various theories and models to identify barriers and facilitators of behavior among which Pender’s health promotion model (HPM) is comprehensive. The HPM states that cognitive-perceptual factors (perceived benefits, barriers, and self-efficacy), and modifying factors (demographic variables, interpersonal influences, and behavioral factors) are considered to interact with each other to shape and influence an individual’s health-promoting behaviors. The approach of this model is self-actualization in order to be healthier (not the absence of disease), so it is an approach-oriented or competence-oriented model [10, 11]. The dimensions of HPM are as follows: Perceived self-efficacy: The individuals’ belief in their ability to organize and implement a specific flow of activity or performance. Perceived barriers: They refer to the individual perception associated with lack of access, lack of suitability, cost, difficulty or time-consuming nature of a particular behavior. Perceived benefits: They refer to action anticipating benefits, psychological visualization of positive or behavior-reinforcing consequences. Interpersonal influences: knowledge about other individuals’ behavior, beliefs, or attitudes. Situational influences: Individuals’ perceptions and cognitions of any situation or context that can facilitate the person’s behavior or prevent it. Commitment to plan of action: A behavioral event that leads a person towards certain behavior in the case of avoiding competitive demands (Fig. 1) [10, 12].

Given that various constructs have been introduced as the strongest predictors of oral-health-related behaviors, including brushing behavior, based on the studies and the above-mentioned model [13, 14], the present study aimed to determine the roles of correlational factors based on the Pender’s health promotion model in brushing behavior of ninth-grade students at urban schools of Gilan in Iran.

## Methods

### Study design and sampling

In the cross-sectional study, the participants included 761 ninth-grade students at urban public schools of Guilan province in the academic year of 2019. The necessary minimum sample size was 501 according to a study by Pakpour et al. [15] in which 30% of ninth-grade students reported that they regularly brushed their teeth by taking into account the 4% error of approximation, and 0.05 type-I error probability. The sample size became 708 according to the loss of data at a maximum of 15% of the samples and the coefficient of plan equal to 1.2.

For familiarity of samples for the entire Guilan province, we selected Rasht, Talesh, Fuman, Anzali, Rudbar, and Langarud counties by a convenience method. For selection of samples, we conducted multi-cluster random sampling and selected Rasht, Lasht-e Nesha, Khoshkebijar, Fuman, Anzali, Rudbar, Rahmatabad, Langarud, and Kumeleh by a random systematic method. At the next stage, we prepared a list of public schools according to the number of students in each county, and selected the schools (*n* = 29) using the simple random sampling, and then selected a class from each school. Given the demographic distribution of relatively equal male and female students, we equally performed sampling of each city. Inclusion criteria: ninth-grade students at public schools of Guilan province, no Orthodontics, presence in the research process, written consent form of students, parents and school principals, and no periodontal disease, which was determined by the oral clinical examination of a health instructor and dentist.

Exclusion criterion: incomplete questionnaire.

### Measures

Data collection tool was a questionnaire consisting of 3 parts, the first part of which included demographic characteristics. This section contained items on city of residence, age, gender, level of study, birth rank, parents job, parental education, number of children in the family, and the economic situation (The economic situation was measured according to asking students how they assessed their economic status. The possible response categories were good, medium and bad). the second part included the health promotion model constructs. This section was contained of seven constructs derived from 62 items. Table 1 presents the number of questions and range of values ​​that can be obtained for these two parts. The last part of the questionnaire included a question about the frequency of brushing per day. Brushing twice a day (favorable behavior) received score 1, and the lower frequency received score 0 (undesirable behavior).

**Table 1:** Description of the structures of the health promotion model in the studied students

Variable	Number of questions	Cronbach’s alpha coefficients	Average	Standard deviation	Range of achievable values	Range of values obtained	Percentage of average score obtained
Activity related affect	8	0.76	30.74	5.34	8–40	8–40	71
Perceived self- efficacy	7	0.85	23.33	6.52	7–35	7–35	58
Perceived barriers of action	13	0.85	31.22	10.14	13–65	13–65	35
Perceived benefits of action	9	0.62	63.45	4.60	9–45	17–45	69
Interpersonal influences	13	0.81	45.08	10.16	13–65	13–65	55
Situational influences	9	0.76	24.54	6.63	9–45	9–45	43
Commitment to plan of action	3	0.79	9	3.21	3–15	3–15	51

We determined validity and reliability of the primary tools according to a questionnaire by Morowati et al. [16], and face validity was surveyed by asking questions from 20 ninth-grade students. They mentioned ambiguities and shortcomings and eventually the research team revised it and made fundamental changes (item impact score = 5).

A panel of 8 experts (3 specialists in epidemiology, 3 specialists in health education,and two dentistry specialists) evaluated questions based on relevance. After removing some questions and making modification, the rates of content validity index, and content validity ratio were 0.85 and 0.80 respectively for all constructs. To investigate the reliability of tool, 40 students (20 girls and 20 boys) completed the questionnaires twice at an interval of a week, and the internal consistency was calculated using the Cronbach’s alpha (Table 1).

### Statistical analysis

We analyzed data in SPSS21. We used descriptive indices of frequency (frequency percentage), mean (standard deviation), or median (range of changes) according to the variable (quantitative and qualitative) and possible distribution (normal and abnormal). We used the multiple logistic regression model to investigate the predictability of demographic characteristics, score of each construct of the model with behavior (desired behavior and at least 2 brushing per day) [5] by controlling the possible confounding variables. We considered *P* < 0.05 as the significance of the test.

## Results

In the present study, 761 students participated most of whom were in the age group of 15 years, and 50.9% of them were boys. 33.4 and 34.4% of fathers and mothers respectively had high school diplomas. 55.3% of fathers were “self-employed” and 79% of mothers were “housewives”. 53.5% of students had two children in their family. 53.2% of students think that the economic situation is ‘medium ‘(Table 2).

**Table 2:** Description of demographic characteristics of the studied students (number = 761 people)

Specifications		Abundance	Percentage
City of residence	Rasht, Khoshkbijar, Lasht Nasha	484	63.6
	Talesh	60	7.9
	Foman	53	7
	Anzali	70	9.2
	Rodbar, Rahmat abad	77	10.1
	Langerod, Komle	17	2.2
Gender	Girl	374	49.1
	Boy	387	50.9
Age	14 and less	191	25.1
	15	473	62.2
	16	97	12.7
Number of children in the family	1	138	18.1
	2	407	53.5
	3	151	20.0
	4 and above	59	7.8
Birth rank	1	387	50.9
	2	255	33.6
	3	76	10.0
	4 and more	42	5.5
Father’s job	Employee	171	22.5
	manual worker	74	9.7
	Free	421	55.3
	the farmer	28	3.7
	Retired	61	8.1
Mother’s job	housewife	601	79
	Employee	92	12.1
	manual worker	5	0.7
	Free	56	7.4
	the farmer	2	0.3
	Retired	4	0.5
Father Education	illiterate	22	2.9
	Primary	104	13.7
	Middle school or high school	199	26.1
	diploma	254	33.4
	Post-diploma, bachelor’s degree and higher	178	23.4
Mother’s education	illiterate	25	3.3
	Primary	112	14.7
	Middle school or high school	202	26.5
	diploma	262	34.4
	Post-diploma, bachelor’s degree and higher	160	21
The economic situation	Bad	17	2.2
	medium	405	53.2
	Good	337	44.3

A total of 48.7% of students brushed their teeth at least once a day and 20.1% twice a day (Table 3). There were statistically significant relationships between the frequency of brushing and gender, and parents’ education level (*P* < 0.001), so that students with more literate parents and female students had more desirable behavior (Table 4).

**Table 3:** Description of the frequency of brushing according to the statements of the studied students

Specifications	Abundance	Percentage
They do not brush at all.	17	2.2
Brush less than once a day or irregularly.	373	49.0
They brush once a day.	218	28.6
They brush twice or more a day.	153	20.1

**Table 4:** Relationship between demographic characteristics and brushing behavior in the studied students

Level		Brush at least 2 times a day		Brush less than 2 times a day		Total	*p*- value
		Abundance	Percentage	Abundance	Percentage		
Gender	Girl	279	74.6	95	25.4	374	0.001
	Boy	329	85.0	58	15.0	384	
Age	14–13	150	78.5	41	21.5	191	0.733
	15	378	79.9	95	20.1	473	
	16	80	82.5	17	17.5	97	
Number of children in the family	1	111	80.4	27	19.6	138	0.958
	2	326	80.1	81	19.9	407	
	3	118	78.1	33	21.9	151	
	4 and above	47	79.9	12	20.3	59	
Birth rank	1	308	79.6	79	20.4	387	0.950
	2	203	79.6	52	20.4	255	
	3	61	80.3	15	19.7	76	
	4 and more	35	93.3	7	16.7	42	
Father’s job	Employee	129	75.4	42	24.6	171	0.230
	manual worker	65	87.8	9	12.2	74	
	Free	336	79.8	85	14.3	421	
	the farmer	24	85.7	4	19.7	28	
	Retired	49	80.3	12	23.3	61	
Mother’s job	housewife	122	76.7	37	23.3	159	0.276
	Non-housewife	485	80.7	116	19.3	601	
Father Education	illiterate	21	95.5	1	4.5	22	0.016
	Primary	93	89.4	11	10.6	104	
	Middle school or high school	160	80.4	39	19.6	199	
	diploma	196	77.2	58	22.8	254	
	Post-diploma, bachelor’s degree and higher	135	75.8	43	24.2	178	
Mother’s education	illiterate	21	77.2	4	16.0	25	0.008
	Primary	98	75.8	14	12.5	112	
	Middle school or high school	172	84.0	30	14.9	202	
	diploma	198	87.5	64	24.4	262	
	Post-diploma, bachelor’s degree and higher	119	85.1	41	25.6	160	
The economic situation	Good	263	78.0	74	22.0	337	0.269
	Medium / bad	343	81.3	79	18.7	422	

The highest mean scores of the behavior-feeling constructs belonged to perceived benefits, perceived self-efficacy, interpersonal influences, commitment to plan of action, situational influences, and perceived barriers respectively (Table 5).

**Table 5:** Description of the structures of the health promotion model in the studied students

Variable	Number of questions	Cronbach’s alpha coefficients	Average	Standard deviation	Range of achievable values	Range of values obtained	Percentage of average score obtained
Activity related affect	8	0.76	30.74	5.34	8–40	8–40	71
Perceived self- efficacy	7	0.85	23.33	6.52	7–35	7–35	58
Perceived barriers of action	13	0.85	31.22	10.14	13–65	13–65	35
Perceived benefits of action	9	0.62	63.45	4.60	9–45	17–45	69
Interpersonal influences	13	0.81	45.08	10.16	13–65	13–65	55
Situational influences	9	0.76	24.54	6.63	9–45	9–45	43
Commitment to plan of action	3	0.79	9	3.21	3–15	3–15	51

Using a multiple linear regression model for determining the relationships of other constructs of the health promotion model with the commitment to plan of action, it was found that 58% of the variance of the dependent variable (commitment to plan of action) was described by the constructs. Based on the multiple linear regression model, the perceived self-efficacy, situational influences, and perceived barriers respectively had stronger significant relationships with commitment to plan of action (Table 6).

**Table 6:** Regression coefficients and 95% confidence interval of students in predicting the commitment to plan of action based on the constructs using multiple linear regression model by Enter method

Variable	Non-standard coefficient (B)		Standard coefficient (B)	T	Probability value	95% confidence interval for regression coefficients (B)		R2
	Coefficient	The standard error				Low limit	upper line	
Constant number	−0.06	0.89		−0.07	0.94	−1.81	1.68	58%
Activity related affect	0.05	0.01	0.09	3.18	< 0.001	0.21	0.09	
Perceived self -efficacy	0.24	0.01	0.49	14.64	< 0.001	0.21	0.27	
Perceived barriers of action	−0.04	0.01	−0.13	−4.38	< 0.001	− 0.63	− 0.02	
Perceived benefits of action	0.00	0.01	0.00	0.22	0.82	−0.03	0.04	
Interpersonal influences	0.01	0.00	0.03	1.27	0.20	−0.00	0.02	
Situational influences	0.10	0.00	0.21	7.38	< 0.001	0.07	0.12	

The commitment to plan of action, self-efficacy, and situational influences were significant according to the evaluation of odds ratio in predicting brushing behavior at least twice a day in students based on the health promotion model constructs using multiple logistic regression analysis by “Enter” method. Based on results of logistic regression and assuming that the other variables of the model were constant, one-unit increase in the score of commitment to plan of action enhanced the odds ratio of brushing behavior by 14% for brushing at least twice a day. One-unit increase in self-efficacy enhanced the odds ratio of brushing behavior by 12% for brushing at least twice a day (Table 7).

**Table 7:** Odds ratio and 95% confidence interval in predicting students’ brushing behavior (at least 2 times per day) based on the health promotion model constructs using multiple logistic regression analysis by Enter method

Variable	Odds ratio	95% confidence interval		*P*
		Low limit	upper line	
Commitment to plan of action	1.143	1.034	1.264	0.009
Activity related affect	0.997	0.946	1.050	0.903
Perceived self- efficacy	1.117	1.056	1.181	< 0.001
Perceived barriers of action	0.978	0.950	1.006	0.122
Perceived benefits of action	0.980	0.931	1.031	0.437
Interpersonal influences	1.016	0.992	1.042	0.194
Situational influences	0.96	0.93	0.99	0.04

## Discussion

The results of the present study indicated that the optimal brushing behavior was 20.1% in students. The result was consistent with studies in Iran that reported the optimal brushing behavior of 17.4 to 18.2% [15, 17]. In other studies conducted abroad, they reported brushing behavior at least twice a day in students in India, Bangladesh, and Nepal from 24 to 37.4% that were close to the present results [18–20] and the behavior was reported to be from 68 to 95.2% in studies by Kırtıloğlu et al. in Turkish students [21], Soroye et al. in Nigeria [22], Vettore et al. in the Brazilian adolescents [23], and 67.2% in a study by Peltzer et al. on students from 26 countries [24], 92.1% in a study by Rimondini et al. in Italian students [25], and 95% in a study by Jensen et al. [26] in different age groups in Sweden and the observed difference could be due to differences in income of countries, parents’ education level, gender, the way of understanding the issues, self-esteem, difference of ethnic groups and migration, cultures, different beliefs and habits in the field of oral health behavior, and integrated education of oral hygiene at schools.

Our findings indicated that students’ good brushing behavior had a statistically significant relationship with parents’ education level; and students, who had parents with higher education, significantly had better brushing behavior. The finding was consistent with studies by Vakili et al. [13], Pakpour et al. [15], who reported a significant relationship between parents’ education levels and good behavior. However, a study by Casanova et al. [27] in Mexican students did not show any significant relationship between parental education levels and frequency of brushing probably due to cultural and social differences in the research group. A possible reason for the high level of good behavior in students with highly educated parents can indicate high health literacy in their parents. Other possible reasons may be that parents are considered as role models for students and positive effects on oral health behaviors; hence, the parents’ roles should be considered in designing the educational interventions.

Our findings indicated that brushing behavior at least twice a day was higher in female students than male students. The results were consistent with most studies on this field [18, 24, 28]. A possible reason for this finding is that women care more about the beauty of their body and appearance, and thus their health than men. Therefore, they pay more attention to their health as an effective factor to increase the beauty and appearance of the body. Understanding belief systems relating to health issues is crucial in any culture for developing health promotion programs in that cultural context.

In the present study, health promotion model constructs were able to describe 58% of the variance of commitment to plan of action. Banaye Jeddi et al., [29] predicted model constructs by 26.4% of the variance of commitment to plan of action. As seen in our study, the predictive power of model constructs was high in commitment to plan of action; and the perceived self-efficacy, situational influences, and perceived barriers were the strongest predictors of commitment to plan of action. In a study by Goodarzi et al. [30] perceived self-efficacy was the strongest predictors, and in a study by Banaye Jeddi et al. [29], the perceived barriers and situational influences were the strongest predictors of the commitment to plan of action. In the present study, the important role of self-efficacy in predicting commitment to plan of action indicated that planning and implementing the educational interventions with an aim to increase self-efficacy could be effective in promoting commitment to plan of action. Given that influencing on barriers is not easily possible in most cases, an educational intervention should target the processes of reducing barriers by identifying real and perceived barriers and paying special attention to the most important ones [31].

According to the prediction of desired behavior, the commitment to plan of action and self-efficacy were significant predictors of behavior, and one-unit increase in the above constructs enhanced the chance of performing the behavior by 14 and 12% respectively. Consistent with results of our study, Zeidi et al. [32], Banaye Jeddi et al. [33], and Mehri et al. [34], reported that the commitment to plan of action was the strongest predictor of oral health behavior. However, results of studies by Morowati et al. [16], Charkazi et al. [35], and Vakili et al. [13], were inconsistent with our study. Different results of the above studies may be due to different economic, social, cultural and age conditions in the research groups. Commitment to plan of action starts up the behavioral event. This commitment leads people towards the behaviors; and the higher commitment to a specific activity increases the possibility of maintaining healthy behavior at the whole time. The commitment to plan of action refers to planning for its change, start, maintenance, and management [10]. Therefore, the more committed you are to planning to perform a behavior regardless of constraints, conditions, and possibilities, the more likely you are to engage in that behavior, and the more successful you are in achieving that goal. Since behavior is a complicated phenomenon and its change is a continuous, permanent, and goal-based process, it is impossible to achieve a logical and purposeful result without relying on the specified and planned commitment that requires knowledge about future opportunities and threats and prediction of the way of facing with them. Commitment to plan of action can lead to desirable behavior when it creates more attractiveness than other behaviors because otherwise other behaviors are preferred. It is worthwhile to consider the above point in designing educational interventions for the research group.

Consistent with our study, findings of many studies emphasize the role of self-efficacy as a significance predictor of oral health behavior [13, 14, 30, 32–36]. Self-efficacy is not about a person’s skills, but about his or her judgment of what he or she can do, considering all the skills he has [10]. Higher self-efficacy creates a positive feeling about the behavior and decreases perceived barriers. Therefore, its increase in students by creating the ability to overcome the existing barriers on the pathway to observance of the oral health can maintain the continuity of desired behavior. In other words, the students’ belief in their personal and inner abilities to overcome barriers is a key factor in the adhering the recommendations of oral hygiene. Given the results of the present study, we suggest a higher focus on the identification of internal and environmental perceived barriers of students in the field of oral health behavior, and then the use of strategies such as verbal persuasion, stimulating and encouraging emotional states and vicarious learning resulting from observing the performance of others to improve students’ self-efficacy.

The research limitations included the use of self-report tools and the cross-sectional nature of study; hence, we could not infer the causal relationships.

## Conclusion

According to the results, the rate of optimal behavior (Brush at least 2 times a day) among Iranian students was low. Commitment to action plan and perceived self-efficacy were the most important constructs in predicting optimal behavior in students. It seems that educational interventions targeting brushing behavior might benefit using the Health Promotion Model to improve adolescents’ oral health.

## Supplementary Information

**Additional file 1.**

## Data Availability

The datasets used and/or analyzed during the current study are available from the corresponding author on request.

## Abbreviations

WHO: World Health Organization
OR: Odds Ratio
SD: Standard Deviation
SPSS: Statistical Package for Social Sciences
CVR: Content validity ratio
CVI: Content validity index
