# Supplementary material for: The role of correlated factors based on Pender health promotion model in brushing behavior in the 13–16 years old students of Guilan, Iran
Source: Ital J Pediatr. 2021 May 12;47:111. doi: 10.1186/s13052-021-01063-y (PMC8117318; doi:10.1186/s13052-021-01063-y)
Supplement: Supplementary file 1 — Additional file 1. [file 13052_2021_1063_MOESM1_ESM.docx]

Additional files1

(questionnaire)

**Please select the appropriate option.**

1- What effect does fluoride have on teeth?

A- It whitens teeth. B- It increases the growth of teeth.

C- It prevents tooth decay. D- It reduces the growth of teeth.

2- Which option is correct for choosing a toothbrush?

A- Rough toothbrush B- Medium toothbrush

C- Soft toothbrush D- The type of toothbrush does not matter.

3- What is dental plaque?

1. It is a kind of toothpaste.

B- It is a black spot on the surface of the tooth.

C- The formation of a layer of microbes and nutrients on the tooth surfaces.

D- The food left between the teeth.

4- What is the most important cause of bleeding gums when brushing?

A- Gum disease B- Tooth decay

C- Tooth calculus D- Improper brushing

5- Which of the following is a sign of tooth decay?

A- The presence of brown or black spots on the teeth B- Sensitivity of teeth to cold or heat

C- Feeling pain when chewing food D- All cases

6- How often should a toothbrush be changed?

A- 1 to 3 months B- 4 to 6 months

C- 7 months to 1 year D- more than 1 year

7- How many times a day is it recommended to brush our teeth?

A- Once in the morning B- Once at night

C- Twice "morning and night" D- More than twice a day

8- How much time should be spent for each brushing?

A- one minute B- two minutes C- three minutes D- 4 to 5 minutes

9- Please specify to what extent you have any of the following feelings while brushing?

| Too much | Very | To some extent | Little | not at all |  |
| --- | --- | --- | --- | --- | --- |
|  |  |  |  |  | 1) Feeling freshness and vitality |
|  |  |  |  |  | 2) Feeling pleasure |
|  |  |  |  |  | 3) Feeling relaxation |
|  |  |  |  |  | 4) Feeling useful |
|  |  |  |  |  | 5) Feeling of increased self-confidence |
|  |  |  |  |  | 6) Feeling a waste of time |
|  |  |  |  |  | 7) Feeling of doing nothing in vain |
|  |  |  |  |  | 8) Feeling bored |
|  |  |  |  |  | 9) Feeling hatred |

10- How confident are you in doing the following activities?

| Always | Often | Sometimes | Occasionally | Never |  |
| --- | --- | --- | --- | --- | --- |
|  |  |  |  |  | 1) I can brush even if I am tired and bored. |
|  |  |  |  |  | 2) I can brush properly. |
|  |  |  |  |  | 3) I can brush every time for the required time (at least 2 minutes) |
|  |  |  |  |  | 4) I can brush even if I have a lot of exams or assignments. |
|  |  |  |  |  | 5) I can brush regularly even if my parents do not remind me. |
|  |  |  |  |  | 6) I can brush regularly even while traveling. |
|  |  |  |  |  | 7) I can brush regularly during the holidays. |

11- To what extent does each of the following options prevent you from brushing your teeth?

| Too much | Very | To some extent | Little | Not at all |  |
| --- | --- | --- | --- | --- | --- |
|  |  |  |  |  | 1) Excessive fatigue |
|  |  |  |  |  | 2) Not having enough time to brush |
|  |  |  |  |  | 3) Lack of familiarity with the benefits of brushing |
|  |  |  |  |  | 4) Doing homework and other homework |
|  |  |  |  |  | 5) Doing activities of interest |
|  |  |  |  |  | 6) Reluctance (reluctance) |
|  |  |  |  |  | 7) Physical problems (colds, headaches, etc.) |
|  |  |  |  |  | 8) Being sad |
|  |  |  |  |  | 9) High cost of toothbrushes and toothpaste |
|  |  |  |  |  | 10) Boredom |
|  |  |  |  |  | 11) Having pain when brushing |
|  |  |  |  |  | 12) Time consuming |
|  |  |  |  |  | 13) Forgetting |

12- Please specify the degree of your agreement or disagreement with the following sentences.

| Strongly disagree | Disagree | Uncertain | Agree | Strongly Agree |  |
| --- | --- | --- | --- | --- | --- |
|  |  |  |  |  | 1) Brushing eliminates bad breath. |
|  |  |  |  |  | 2) Good oral health increases social credibility. |
|  |  |  |  |  | 3) Brushing makes a smile more beautiful. |
|  |  |  |  |  | 4) Brushing is not effective in preventing tooth decay. |
|  |  |  |  |  | 5) Good oral condition can be effective in mental health (having a good mood). |
|  |  |  |  |  | 6) Brushing has no effect on eliminating or reducing gum disease. |
|  |  |  |  |  | 7) Oral health is effective in preventing heart disease. |
|  |  |  |  |  | 8) Observing oral health makes me better able to eat. |
|  |  |  |  |  | 9) The cost of prevention (such as buying toothbrushes and toothpaste and visiting the dentist regularly) is less than the cost of treatment. |

13­- To ­what extent do the following people encourage you to brush your teeth?

| Completely | Very | To some extent | Little | Not at all |  |
| --- | --- | --- | --- | --- | --- |
|  |  |  |  |  | Father |
|  |  |  |  |  | Mother |
|  |  |  |  |  | Brothers and sisters |
|  |  |  |  |  | Friends |
|  |  |  |  |  | Health caregiver |
|  |  |  |  |  | Teachers and parents of the school |

14- What is the situation of brushing of your family members?

| Always | Often | Sometimes | Occasionally | Never |  |
| --- | --- | --- | --- | --- | --- |
|  |  |  |  |  | Father |
|  |  |  |  |  | Mother |
|  |  |  |  |  | Brothers and sisters |

15. How effective is brushing the following people in your brushing?

| Completely | Very | To some extent | Little | Not at all |  |
| --- | --- | --- | --- | --- | --- |
|  |  |  |  |  | Father |
|  |  |  |  |  | Mother |
|  |  |  |  |  | Brothers and sisters |
|  |  |  |  |  | Friends |

16. What is the source of information for your toothbrush?

| Completely | Very | To some extent | Little | Not at all |  |
| --- | --- | --- | --- | --- | --- |
|  |  |  |  |  | Book |
|  |  |  |  |  | Journal |
|  |  |  |  |  | Internet |
|  |  |  |  |  | Social networks (Telegram - WhatsApp - Instagram) |
|  |  |  |  |  | Family |
|  |  |  |  |  | Friends |
|  |  |  |  |  | Dentist |
|  |  |  |  |  | Other health personnel |
|  |  |  |  |  | radio and TV |

17. To what extent are you committed to the following behaviors?

| Always | Often | Sometimes | Occasionally | Never |  |
| --- | --- | --- | --- | --- | --- |
|  |  |  |  |  | 1. Brushing is so important to me that I set a specific time for it. |
|  |  |  |  |  | 2. I am committed to my brushing schedule. |
|  |  |  |  |  | 3. I encourage others to brush |

18. How do you do the following behaviors?

1- How often do you brush your teeth? Write its number.

Daily ............. Weekly ............. Monthly .............. Annual ...... ........ never............

2- How often do you floss? Write its number.

Daily ............. Weekly ............. Monthly .............. Annual ...... ........ never............

3- When was the last time you referred to the dentist?

1. I have not referred to the dentist
2. More than one year ago
3. One year ago
4. The last six months
5. Less than the last six months

4- If you refer to the dentist, what is the most important cause of your referral to the dentist?

1. Regular examining of my teeth
2. Force of parents
3. Having tooth pain or problems in the gums
4. Other causes
